# Method of Applying Chloride of Zinc and Nitrate of Silver

**Published:** 1871-10

**Authors:** 


*Method of Applying Chloride of Zinc and Nitrate of Silver.*—Dr. H. Judd in a paper on diseases of the gums and their treatment, read before the District Dental Society, Buffalo, N. Y., gives the following method of reaching the margins of the processes, or the periosteal structures at that point when they are reflected from the surface of the alveolar process into the alveolus, the great point being to reach the margins of the alveolus itself with the remedial agent employed:

"My method of reaching the seat of the affection with the medicine is as follows:—I make a short silver blade about three-fourths of an inch long and half an inch wide, of the thickness of a common silver plate, say number 26 of the guage plate, and solder on another piece of the plate to about one-fourth of an inch of it at one end upon both sides, so that one end will be three thicknesses of the plate. The silver being readily bent to any curve desired, it is more easily introduced between the teeth when this is desirable. The thick end of the blade can be easily grasped by a pair of plyers or any other instrument which will suffice to carry it where it is desired. If you wish to use the nitrate of silver, place a little of the crystals upon the blade near the end, and hold it for a moment in the flame of a spirit lamp. It will fuse and be converted at once into a glassy fluid, which upon removing it from the lamp and allowed to cool, will be found to adhere closely to the blade, and can then be easily carried up under the edge of the gums and brought in contact with the periosteum covering the margin of the alveolus, thus making the application directly to the place where it is required. If chloride of zinc is to be used, it can be done in the same way, although if it is heated in this way it does not cling very tenaciously to the blade, still if the heat applied be not too great it will be attached sufficiently to the blade so that it can be carried up under the margins of the gums with little difficulty. Two or three careful applications a week of one of these agents is all that is required, aud this treatment should be persistently continued until the gums become hard and firmly closed around the necks of the teeth. It is my opinion that a large majority of cases of this kind, if taken before the teeth have become much loosened, can in this way be cured, but it requires great care afterwards to prevent a return of the disease."

